# Yop1 stability and membrane curvature generation propensity are controlled by its oligomerisation interface

**DOI:** 10.1042/BCJ20240190

**Published:** 2024-10-11

**Authors:** Anu V. Chandran, Daniel Álvarez, Stefano Vanni, Jason R. Schnell

**Affiliations:** 1 Department of Biochemistry, University of Oxford, South Parks Road, Oxford OX1 3QU, U.K.; 2 Department of Biology, University of Fribourg, Fribourg, Switzerland; 3 Departamento de Química Física y Analítica, Universidad de Oviedo, Oviedo, Spain; 4 Swiss National Center for Competence in Research (NCCR) Bio-inspired Materials, University of Fribourg, Chemin des Verdiers 4, CH-1700 Fribourg, Switzerland

**Keywords:** DP1, hereditary spastic paraplegia, membranes, reticulon homology domain, Yop1

## Abstract

The DP1 family of integral membrane proteins stabilize high membrane curvature in the endoplasmic reticulum and phagophores. Mutations in the human DP1 gene REEP1 are associated with Hereditary Spastic Paraplegia type 31 and distal hereditary motor neuropathy. Four missense mutations map to a putative dimerization interface but the impact of these mutations on DP1 structure and tubule formation are unknown. Combining biophysical measurements, functional assays, and computational modeling in the context of the model protein Yop1, we found that missense mutations have variable effects on DP1 dimer structure and *in vitro* tubulation activity, and provide mechanistic insights into the role of DP1 oligomerisation on membrane curvature stabilization*.* Whereas the mutations P71L and S75F decreased dimer homogeneity and led to polydisperse oligomerization and impaired membrane curving activity, A72E introduced new polar interactions between subunits that stabilized the Yop1 dimer and allowed robust tubule formation but prevented formation of more highly-curved lipoprotein particles (LPP). The introduction of a BRIL domain to the cytoplasmic loop of A72E rescued LPP formation, consistent with a requirement for dimer splaying in highly curved membranes. These results suggest that the membrane curving activity of DP1 proteins requires both dimer stability and conformational plasticity at the intermolecular interface.

## Introduction

Intracellular membranes such as the endoplasmic reticulum (ER) and phagophores are morphologically distinct and contain regions characterized by high membrane curvature. Highly curved membranes are found in ER in tubules and at the edges of sheets, and in the rims of phagophores. The proteins responsible for stabilizing these highly curved membranes are members of the reticulon and DP1 families found exclusively in eukaryotes [1–5].

The DP1 proteins can be divided into two subfamilies depending on whether they contain three or four α-helical transmembrane domains. The human REEP1–4 and yeast Rop1/Yep1 belong to the former subfamily, and the human REEP5–6 and Yop1 to the latter. Members of both families have been shown to curve membranes in cells and *in vitro* [4,6,7], indicating that the first transmembrane helix is dispensable for this function. The remaining three transmembrane helices, as well as a C-terminal amphipathic helix are required for efficient membrane curvature stabilization [4,6–9].

DP1 proteins form stable dimers [7], and possibly weaker, higher order oligomers [6,10]. Genetic mutations in the human DP1 protein REEP1 that are associated with autosomal dominant hereditary spastic paraplegia type 31 (SPG31) [11–17] and distal hereditary motor neuropathy type V (dHMN5) [18] are found at this dimer interface [19,20]. To better understand the role of specific residues within the membrane curving domain of DP1 proteins, and to identify potential structural and functional mechanisms underlying SPG31 pathology, we investigated the protein stability, dimerization, and *in vitro* tubule formation of three SPG31-associated substitutions in the Yop1 sequence context.

**Figure 1.**
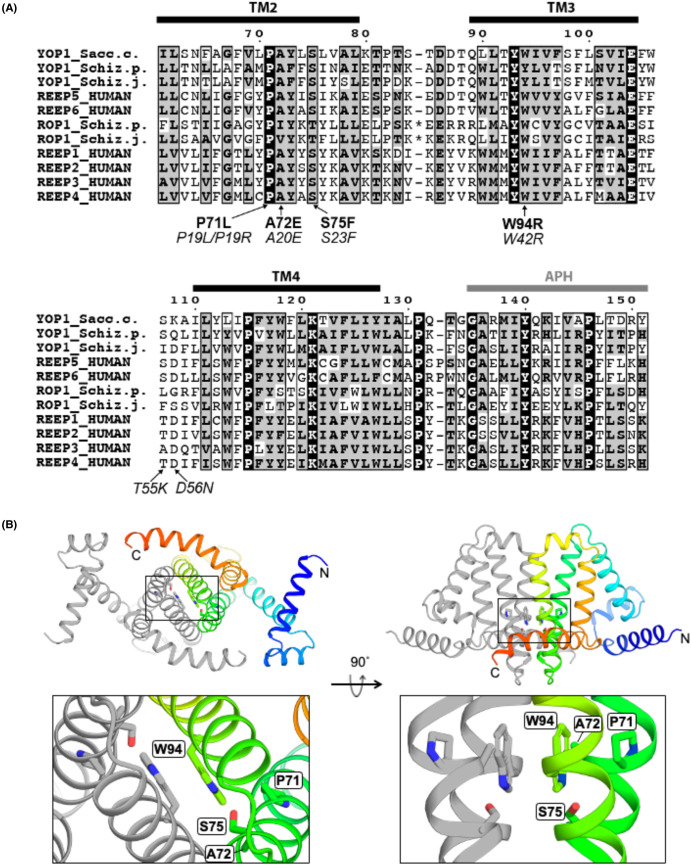
Multiple sequence alignments and the predicted dimer structure. (**a**) Sequence alignment of human REEPs and the previously characterized yeast proteins Yop1 and Rop1. The Yop1 transmembrane helix 2 (TM2) mutations P71L, A72E, and S75F and the transmembrane helix 3 (TM3) mutation W94 are indicated, as well as the REEP1 missense mutations associated with SPG31 (italics). An asterisk indicates 20 residue insertions present in the cytoplasmic loops of *Schizosaccharomyces pombe* and *Saccharina japonica* Rop1 proteins. The sequences were aligned in T-Coffee and formatted by ESPript 3.0. Residues at both the N- and C-termini are omitted. (**b**) Cartoon overview of the Yop1 dimer structure predicted by AlphaFold-Multimer, with the sidechains of P71, A72, S75, and W94 shown as sticks, oxygen in red, and nitrogen in blue. One subunit is shown in gray and the other in blue to orange colorscale from N- to C-terminus. The residues 157–180 predicted to be unstructured have been omitted. Left-hand structures are viewed into the plane of the membrane, and right-hand structures are viewed from within the membrane bilayer. Bottom structures are zoomed into the dimer interface with positions homologous to SPG31-associated missense mutation sites indicated. The amphipathic helix residues 134–151 have been removed from the lower right-hand structure for clarity.

Yop1 is a yeast DP1 protein with high sequence conservation with human REEPs that is sufficient to stabilize lipid membrane tubules in cells and *in vitro* [6–8]. Sequence alignment between all six human REEPs and the characterized yeast DP1 proteins indicate strong amino acid conservation across transmembrane helices two to four and the C-terminal amphipathic helix (Figure 1a). The high sequence conservation of the DP1 transmembrane domain from yeast to humans and the ability of both Yop1 and REEP1 to stabilize high membrane curvature in cells and *in vitro* suggest that Yop1 can provide insights into the mechanisms by which DP1 proteins function.

We found that introduction of REEP1 SPG31-associated substitutions in the Yop1 context results in changes to both oligomerization and membrane curving activities. Structural modeling and molecular dynamics (MD) simulations of Yop1 variant dimers indicate that mutations affect intramolecular or intermolecular interfaces to different extents, and that whereas the bulky P71L and S75F substitutions introduce steric clashes to the intra- and inter-subunit packing, resulting in polydisperse oligomerisation, the substitution A72E introduces new interfacial polar contacts that stabilize the dimer but reduce membrane curving activity. The A72E variant could be made to form exclusively high curvature spherical particles by introduction of steric clash at the cytoplasmic loop nearest the dimer interface, suggesting that A72E stabilizes a fixed dimer interface at the expense of subunit rotation that is required for stabilizing higher membrane curvature.

## Results

### Disease-associated mutations cluster at the DP1 dimer interface

Eight missense mutations at seven positions in REEP1 are associated with SPG31. The pathogenic significance of the mutations T55K, D56N and L107P is unclear [13–15] and these mutation sites are weakly conserved outside of higher eukaryotes (Figure 1a). The remaining five missense mutations (P19L, P19R, A20E, S23F and W42R) at more highly conserved positions and have been shown to affect ER localization in REEP1 [11,14,15,17,18].

Yop1 and other DP1 proteins form dimers but may also form higher order oligomers of dimers in cells as a scaffold for stabilizing curved membranes [7,10,21,22]. AlphaFold2 models of dimeric Yop1, REEP5 and REEP6 [19,20] indicate that the second and third transmembrane domains are at the putative dimer interface (Figure 1b and Supplementary Figure S1), consistent with chemical cross-linking studies in lipid bilayers [7,10]. Homologous interfaces are seen at the first and second transmembrane helices of REEPs 1–4, which lack the first transmembrane domain (Supplementary Figure S1). In all structures, the positions equivalent to Yop1 residues 71, 72, 75 and 94 are clustered at or near to the dimer interface, and likely define a functional hotspot (Figure 1b and Supplementary Figure S1).

Among the characterized yeast DP1 proteins, Yop1 has a dimer interface that is most similar in sequence to the human REEPs. Using the Yop1 numbering, P71 and W94 are two of the most strongly conserved residues in all DP1 proteins (Figure 1a). The Yop1 A72 and S75 are identical in human REEPs although amino acid identities at these positions are somewhat less conserved than P71 and W94. In all, 59 currently reviewed Swiss-Prot entries for the DP1 family, 71% of the amino acids at position 72 are alanine but several other nonpolar sidechains are also found here, including leucine (14%) and glycine (7%). Position 75 is restricted to small polar residues including serine, cysteine and threonine.

### Stability and biophysical characterization of Yop1 variants

To investigate the effects of missense mutations, Yop1 genes with P71L, A72E, and S75F were produced for expression in *Escherichia coli*. Attempts also were made to produce a sample of the W94R Yop1 variant but this construct failed to express at detectable levels. The remaining Yop1 variants were purified and reconstituted into *n*-dodecyl-β-d-maltoside (DDM) detergent. Preparative size exclusion chromatography (SEC) indicated that the P71L variant, and to a lesser extent S75F, were prone to aggregation (Supplementary Figure S2). In contrast, A72E exhibited little or no aggregation tendency, and was more conformationally homogenous than wildtype in the final gel filtration purification step.

**Figure 2.**
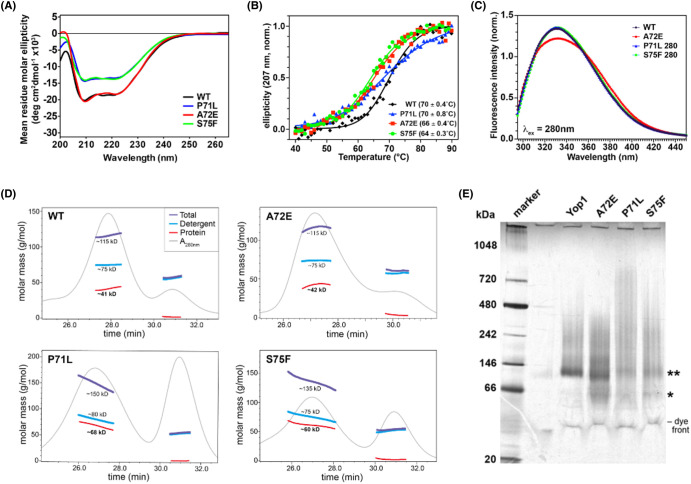
Biophysical characterization of Yop1 variants. (**a**) Circular dichroism (CD) mean residue molar ellipticity of the Yop1 variants. (**b**) Thermal stabilities (*T*_m_) determined from the normalized CD signal at 207 nm. The *T*_m_ values indicated in parentheses were determined by fitting the data shown (40–90°C). (**c**) Tryptophan fluorescence emission spectrum for the Yop1 variants. The excitation wavelength was 280 nm. Emission intensities were normalized by area under the curve. (**d**) SEC-MALS of Yop1 with either wildtype sequence (WT) or one of the indicated transmembrane domain substitutions. The molar masses are colored as indicated for WT and overlayed with the absorbance at 280 nm (arbitrary intensity). The total (detergent and protein), detergent, and protein masses were estimated from the values at the maximum elution peak height. (**e**) Blue native gel electrophoresis of Yop1 wildtype and variants reconstituted into DDM micelles. Protein monomer (*), protein dimer (**), and dye front bands are indicated at right.

Circular dichroism (CD) spectra of all constructs showed similar profiles that were consistent with an α-helical protein (Figure 2a). P71L and S75F exhibited decreased molar ellipticities, which may be partly due to loss of soluble protein during the course of the experiments. The thermal unfolding midpoints indicated that the A72E and S75F variants were destabilized by ∼5°C relative to wildtype (Figure 2b). The unfolding midpoint for P71L was unchanged from wildtype but the slope of its melting curve was half that of wildtype (Hill slope values of 8.2 ± 0.7 and 16.1 ± 1.4, for P71L and wildtype, respectively), indicating greater heterogeneity that is possibly related to oligomerisation (see below).

Based on the TM2-TM3 packing in the structural models (Figure 1b), the A72E substitution should increase the polarity of the W94 indole ring, resulting in a shift in the fluorescence emission spectrum to longer wavelengths. The measured emission spectrum for P71L and S75F were essentially identical with that of wildtype Yop1, with emission maxima of 330–331 nm (Figure 2c). The maximum emission wavelength for A72E was nominally shifted to 332 nm but a red-shifted shoulder was also observed, consistent with a tryptophan in a more polar environment. Besides W94, Yop1 contains two other tryptophan residues at positions 106 and 118. However, W106 and W118 are at the luminal ends of TM3 and TM4, respectively, and therefore predicted to be in a distant membrane plane. We conclude that the red-shifted emission is that of W94, and consistent with the TM2 and TM3 intra-subunit packing that places W94 near residue 72.

### Transmembrane domain substitutions perturb Yop1 oligomerization

The oligomerization of single-site variants and wildtype Yop1 was evaluated first by SEC-MALS. The data for wildtype and A72E were consistent with a discrete dimer (41–42 kDa measured compared with a theoretical mass of 40.6 kDa) whereas data for P71L and S75F were indicative of polydisperse oligomerization [23], and ranged in sizes from ∼55 to 75 kDa (Figure 2d). The oligomerization of the P71L variant appeared to be most perturbed compared with wildtype, as indicated by its large average weight determined by SEC-MALS.

The oligomerization properties of the variants were then evaluated using Blue native gel electrophoresis to determine whether the P71L and S75F variants retained the ability to form discrete dimers. Blue native gels indicated a dimer band for all constructs. P71L also exhibited a smeared band to very large molecular mass species (Figure 2e).

### Changes in oligomerization correlate with changes in membrane curvature generation propensity

**Figure 3.**
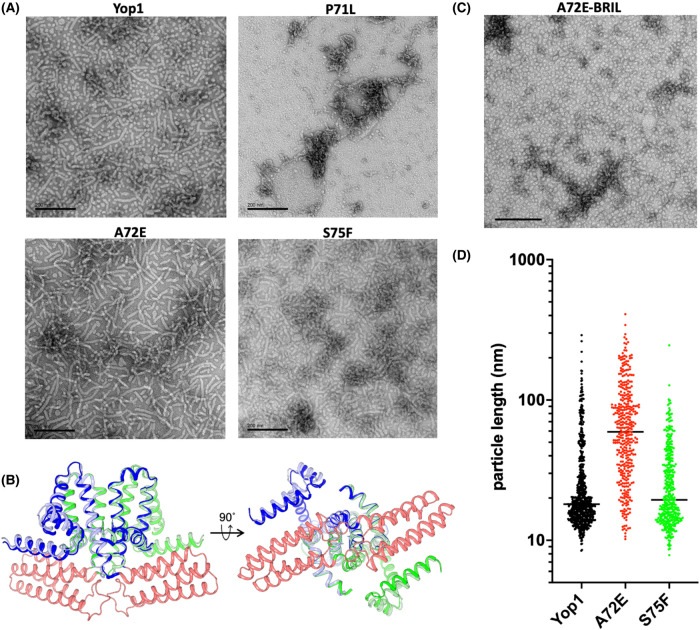
In vitro tubulation assays. (**a**) *In vitro* membrane tubule formation of full-length Yop1 variants using *E. coli* polar lipid extract. Samples were prepared at the same protein and lipid concentrations. (**b**) Alpha-Fold multimer structure prediction for the A72E-BRIL construct dimer. The Yop1 sequence for each subunit in the A72E-BRIL dimer is shown in green or blue, and the BRIL sequence in red. For comparison, the wildtype Yop1 structure is aligned with A72E-BRIL and each subunit shown in light blue or light green. (**c**) *In vitro* membrane tubule formation of Yop1 (left) and A72E-BRIL (right). The membrane tubule assays for wildtype Yop1 and A72E-BRIL were prepared side-by-side and with the same protein and lipid concentrations. (**d**) Maximum end-to-end distances for all unambiguously imaged particles produced with Yop1 (684 particles) and the variants A72E (384 particles) and S75F (423 particles). Horizontal bars indicate the median particle lengths.

The abilities of Yop1 variants to form membrane tubules *in vitro* are shown in Figure 3a. Wildtype Yop1 reconstituted into *E. coli* polar lipid extract showed a mixture of tubules and small spherical vesicles. The spherical particles have a small diameter of 15–20 nm and are likely the same monolayered lipoprotein particles (LPP) observed by Wang et al. [7]. S75F formed tubules with a similar distribution of tubules and spherical particles to wildtype Yop1 but with an increased variation in apparent curvature that was most clearly seen in the LPPs (Figure 3a). In contrast, the P71L variant was significantly impaired in membrane tubule formation, with significant aggregation observed and no clear examples of regular tubular or LPP particles.

The A72E variant produced many tubular particles comparable in length to the longest tubules observed with wildtype Yop1. In contrast with wildtype Yop1, however, A72E produced few spherical particles, indicating that A72E was unable to form LPPs (Figure 3a,d). The high solubility and homogeneity of the A72E variant led to crystallization trials, including the generation of an A72E construct in which the thermostabilized cytochrome b_562_ RIL (BRIL) [24] was inserted into the cytosolic loop of A72E between residues S84 and T85. AlphaFold-multimer predicted the domain insertion could be accommodated within the dimer model (Figure 3b). Yields for the A72E-BRIL construct were comparable to those of A72E but formed exclusively LPPs instead of the tubules observed predominantly with the A72E alone (Figure 3c). The BRIL insertion therefore rescued the ability of A72E to stabilize highly curved LPPs.

### Disease-associated mutations alter molecular interactions at the dimer interface

**Figure 4.**
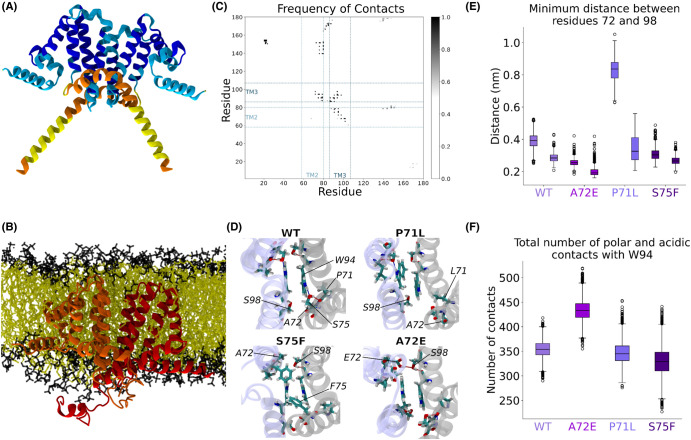
Molecular dynamics simulations of Yop1 variants. (**a**) AlphaFold-Multimer model of dimeric Yop1. (**b**) Atomistic molecular dynamics simulations of the Yop1 dimer model in a DOPC bilayer. (**c**) Intermolecular contact map showing extensive TM3-TM3, and TM2-TM3 interactions, as well as additional contacts between TM2 and the APH. (**d**) View into the plane of the membrane showing the dimer interface for snapshots of the WT, P71L, A72E and S75F simulations. Hydrogen bonds between hydroxyl O–H and carbonyl O groups are shown in red and those between amide (N–H) and carbonyl (O) are in blue. Whereas WT, P71L and S75F show only intramolecular hydrogen bonds, A72E (lower right) forms a new hydrogen bond across the dimer interface which is donated from the carboxylic acid of A72E to the hydroxyl oxygen of S75 from the other monomer. Residues 72 and 98, which are close to each other in all interfaces but one of the P71L mutant, are indicated in all variants. (**e**) Minimum distances between residues 72 and 98 in the 300–400 ns time interval for the four Yop1 variants. (**f**) Number of polar and acidic contacts with W94 (threshold of 5 Å) for the four variants, during the entire simulations.

To investigate possible mechanisms for the structural and functional differences observed between the variants, we next performed atomistic MD simulations. To do so, AlphaFold-multimer models of dimeric Yop1 variants were inserted into planar DOPC bilayers and atomistic simulations were run for 400 ns (Figure 4a,b). The disposition of the two subunits of Yop1 in the dimer was consistent with the proposed transmembrane model [7,10,19]. To validate this model, we simulated the dimer with NMR chemical shift-derived backbone ψ and φ angle restraints [8] (*k* = 1000 kJ/mol) for 50 ns and compared it to the final wildtype structure. The regions of helicity and overall packing of the structures were similar (backbone heavy atoms RMSD of 2.67 Å), confirming the quality of the model (Supplementary Figure S3).

The dimer interface, mainly consisting of contacts from TM3 to TM3 and TM2 to TM3 (Figure 4c), was maintained throughout the simulations. The mutation sites were positioned at the center of the transmembrane domain interface and formed a small, localized core. A distinct feature of the intermolecular interface was the packing of the W94 indole rings, which remain stacked together in an antiparallel orientation at the symmetry axis (Figure 4d).

The simulations revealed the formation of several intramolecular polar bonds within the otherwise hydrophobic core of the wildtype dimer interface (Figure 4d). The hydroxyl group of S75 formed an intramolecular hydrogen bond with the W94 indole ring as well as with the carbonyl oxygen of P71 (Figure 4d). The position of the pyrrolidine ring of P71 also appears to be important, as it located in a parallel orientation between the highly conserved aromatic rings of W94 and Y93. Although the introduction of the S75F and P71L mutations in our simulations initially breaks the antiparallel orientation of the W94 indole rings (Supplementary Figure S4), it is eventually recovered throughout the simulations. However, the P71L variant forms this interaction at the expense of disrupting one of the two TM2–TM3 interfaces (Figure 4e and Supplementary Figure S5), having a larger effect in this region in comparison with the S75F variant, which maintains an interface very similar to wildtype.

The A72E variant also preserved the dimer interface, but the introduced glutamic acid formed a new intermolecular hydrogen bond across the subunit interface to S98 (Figure 4d), which results in a tighter TM2-TM3 packing (Figure 4e). Despite the presence of this new hydrophilic site in the middle of the lipid bilayer, the dimer indole rings remained stably parallel throughout the simulations (Supplementary Figure S4). However, the A72E substitution increased the polarity of the W94 region (Figure 4f), which may contribute to the observed red-shifted tryptophan fluorescence (Figure 2c).

## Discussion

In this study, we have investigated a set of SPG31-linked missense mutations in the context of the well characterized Yop1 protein to gain insights into the structural elements necessary for DP1 membrane curving activity. The mutation sites are strictly conserved between the human REEP1 and yeast Yop1, and they are predicted to be clustered at the homodimer interface. The DP1 dimer is stable in detergent or lipids, and it is likely required for membrane curving [7].

Optimal interhelical packing and a hydrogen bond appear to stabilize the intramolecular arrangement between TM2 and TM3, which are disrupted by the substitutions at P71 and S75. The P71L substitution was the most detrimental, resulting in polydisperse oligomerization, and with little or no evidence for tubule formation. Prolines are strictly conserved at this position, and structural predictions place P71 in TM2 at the intramolecular interface with TM3, and packing against another highly conserved residue, Y93. It is proposed that P71L disrupts the packing of these transmembrane helices, leading to non-specific transmembrane helix interactions.

The S75 sidechain is predicted to be partly at the intermolecular dimer interface and partly in the intramolecular TM2/TM3 interface. Notably, the prediction indicates a hydrogen bond from S75 to W94. Only serine, threonine, and cysteine are found at position 75 in DP1 homologs, suggesting that this interaction is functionally important. The effects of the S75F mutation in the Yop1 context were intermediate, with stability and oligomerization homogeneity intermediate to wildtype and P71L. While the S75F variant was able to form tubules and LPPs in a similar proportion to wildtype Yop1, there was greater variation in particle sizes, indicating at least modest effects on membrane curvature stabilization.

In DP1 structure predictions, the highly conserved W94 indoles are at the dimer interface close to the center of the membrane bilayer, and their parallel-displaced orientation is consistent with an energetically favorable π-π stacking interaction [25]. The W94R variant of Yop1 could not be expressed in *E. coli*, which is perhaps not surprising given it would place two positive charges next to each other in the hydrophobic core of the protein, and such a mutation would very likely also have severe effects on dimer structure and stability.

Unexpectedly, the A72E substitution, which also places a highly polar sidechain near the middle of the membrane bilayer, caused only minor changes to stability and had no effect on the ability of Yop1 to form homogenous dimers. Yet the A72E variant was functionally distinct, forming almost exclusively tubules and only a few LPPs that tended to be larger and more irregular. It has been shown previously that LPPs, which have very high membrane curvature, are more likely to form at high protein-to-lipid concentrations, suggesting that conversion to LPPs correlates with membrane curving activity [7]. Therefore, the failure of the A72E variant to form LPPs could indicate that the variant is functionally impaired. The structure of the A72E variant of Yop1 is unlikely to be very different to that of wildtype in detergent and in tubules, given that it is stably dimeric in detergent and forms tubules morphologically similar to that of wildtype. In addition, MD simulations of the A72E variant and wildtype in lipid bilayers indicated a stable subunit structure, and the detection of red-shifted tryptophan fluorescence in A72E was consistent with position 72 proximal to W94.

Rather than significant changes in subunit conformation and stability, the inability of A72E to convert tubules to the more highly curved LPPs, and the rescue of this activity by the BRIL insertion could be explained by a functional requirement for dimer splaying that was proposed by Wang et al. [7] to account for how DP1 proteins stabilize membranes with different curvatures. The dimer interface is relatively flat and approximately centered at the stacked W94 indole rings, which might allow for subunit rotation and dimer splaying. In the structural modeling of Yop1, the glutamic acid introduced a polar contact to S98 across the dimer interface, which we propose explains the high homogeneity of the A72E variant. At the same time, this additional intermolecular bond would restrict any interdimer rotation required to convert tubules to LPPs. Depending on the geometry and direction of the splay, dimer rotation may also be disfavored by the exposure of the carboxyl group to the lipid bilayer, since position 72 is at the interface edge.

The BRIL insertion to the cytosolic loop connecting TM2 and TM3 rescues the ability of A72E to convert tubules to LPPs. The cytosolic loops in Yop1 and REEPs are ∼5–6 amino acids-long and close in space in the dimer models. The BRIL insertion can be accommodated within the dimer conformation seen for wildtype and A72E Yop1, maintaining TM2/TM2 and TM3/TM3 intermolecular helix crossing angles of 40–45°. However, the insertion likely increases the propensity for splay by offsetting the increased energy of dimer rotation with the introduction of steric clashes for more parallel dimer arrangements.

We note that the hydroxyl at position 98 in Yop1 is not strictly conserved and the equivalent position in REEP1 is an alanine. Apart from serine, only the small amino acids glycine and alanine are found at this position, which may permit the carboxyl group to make interhelical interactions to backbone groups near position 98, but A72E substitution may be more disruptive to dimer assembly in the REEP1 context.

Additional mechanisms, such as mislocalization, are likely involved in the pathogenesis of SPG31 and dHMN5 in the cellular context [11,14,15]. Nonetheless, our results suggest that all of the variants studied here have the potential to alter the dimerization interface, which could contribute to the dominant phenotype observed in SPG31. Moreover, we conclude that dimerization in DP1 proteins is mechanistically linked to membrane curving activity, and alterations in membrane curving activity can arise from both changes in dimer stability and changes in dimer rotation.

## Materials and methods

### Cloning and mutagenesis

A pCold I vector (Takara Bio) containing the full length Yop1 gene from *Saccharomyces cerevisiae* (Uniprot ID: Q12402) was modified using Gibson Assembly [26] to add an N-terminal (His)_9_ tag followed by a Twin-Strep-tag and a TEV cleavage site. The P71L, A72E, S75F, and W94R variants were generated via site-directed mutagenesis using the GeneArt system (ThermoFisher), and confirmed by sequencing (Source BioScience).

### Protein expression and purification

Native Yop1 and all variants were overexpressed and purified using the following protocol. All bacterial agar plates and liquid cultures contained ampicillin at 100 µg/ml (MP Biochemicals). The plasmid containing the gene of interest was transformed to *E. coli* C43 cells (Sigma–Aldrich). The next day, a starter culture of 250 ml Luria Broth media was inoculated from a single colony and grown overnight at 37°C in a shaker incubator. This overnight culture was diluted into Terrific Broth, supplemented with 1% glucose, to an OD_600_ of 0.05, and this was used as the secondary culture for protein overexpression. The secondary culture was grown at 37°C until an OD_600_ of ∼0.8. At this stage, the culture was cold shocked on ice for 20 min, followed by addition of 1 mM isopropyl β-d-1-thiogalactopyranoside (Fluorochem) to induce protein expression. The cells were incubated at 15°C for 16 h with shaking at 180 rpm and harvested the next day by centrifugation at 6000 rpm (JLA-8.1 rotor) for 15 min. All steps from this point were carried out at 4°C unless otherwise specified.

The harvested cells were resuspended in Buffer A (50 mM HEPES pH 8.0, 300 mM NaCl, 20 mM imidazole, 5% glycerol) and homogenized using a Dounce homogenizer. One tablet of cOmplete Protease Inhibitor (Roche) per 50 ml was added to the resuspended cells to prevent proteolysis. DNase I (Sigma–Aldrich) was added to reduce sample viscosity and the cells were lysed by passing the sample through a cell disrupter (Constant systems, LTD) twice at a pressure of 30 000 psi. The whole cell lysate was centrifuged at 8000 rpm (JLS-16.250 rotor) for 20 min to remove cell debris. The supernatant was then ultra-centrifuged at 45 000 rpm (45 Ti Rotor) for 2 h to collect the membrane fraction. The supernatant was discarded, and the membrane fraction resuspended in Buffer A containing 1% *n*-dodecyl-β-d-maltoside (DDM; Anatrace) and incubated overnight at 4°C with stirring to extract the protein from membranes. The next day, insoluble material was pelleted by centrifugation at 18 000 rpm (JA-25.50 rotor) for 1 h. The supernatant containing the detergent solubilized protein was passed thrice through a 10 ml Strep-Tactin® resin (IBA Life sciences) pre-equilibrated with Buffer A containing 0.05% DDM. Nonspecific binding was reduced with a column wash using Buffer A (+0.05% DDM), and protein eluted using Buffer A (+0.05% DDM) containing 10 mM desthiobiotin. The N-terminal (His)_9_ and Twin-Strep-tag were cleaved by adding an excess of TEV protease to the pooled fractions and dialyzing overnight against 25 mM HEPES at pH 8.0, 300 mM NaCl, 5% glycerol, and 5 mM DTT. The cleaved tag, uncleaved tagged protein, and the TEV protease were removed from the sample by passing thrice through a 5 ml immobilized-metal affinity resin (cOmplete His-Tag Purification Resin; Roche) pre-equilibrated with Buffer A (+0.05% DDM). The flow-through from the column was pooled and concentrated in a 30 kDa MWCO Amicon Ultra-15 Centrifugal Filter (Millipore).

SEC was carried out at room temperature using a Superdex S200 10/300GL Increase column mounted on an ÄKTA Pure chromatography system (GE Healthcare). The SEC buffer contained 20 mM HEPES at pH 7.5, 150 mM NaCl and 5% glycerol. A flow rate of 0.75 ml/min was used and 500 µl peak fractions corresponding to the main peak (at ∼12–14 ml elution volume) were collected, flash frozen in liquid N_2_, and stored at −80°C. Protein purity was confirmed by SDS–PAGE and concentration determined from the absorbance at 280 nm and theoretical extinction coefficients.

### Yop1 lipid tubulation assay

Yop1 tubulation assays were carried out similar to previously published studies [8,27]. Yop1 and variant protein samples (0.25 mg/ml) were mixed in 1:1 mass ratio with *E. coli* Polar Lipid Extract (Avanti Polar Lipids) resuspended in reconstitution buffer (25 mM HEPES pH 7.0,150 mM KCl, 5% Glycerol, 1 mM EDTA, 1% DM). The protein, lipid and detergent solution was kept on ice for 30 min. Bio-beads SM-2 Resin (Bio-Rad) was added to the sample in small increments over three days while incubating at room temperature in a rotating wheel.

Membrane-reconstituted Yop1 samples were applied to glow-discharged carbon layered copper grids (carbon support films, 200 mesh 3 mm copper grids, TAAB Laboratories) and incubated for 2 min before blotting with filter paper. Grids were then stained with 20 µl of 2% uranyl acetate for 10 s, blotted with filter paper, and allowed to dry at room temperature for a few minutes. Images were recorded on a TEM Philips Tecnai 12 (120 keV) electron microscope at a magnification of 49 000×. The maximum end-to-end particle lengths were quantified using Fiji [28].

### SEC-MALS

Protein samples in 20 mM HEPES at pH 7.5, 150 mM NaCl, and 5% glycerol were concentrated to between 1 and 1.4 mg/ml, and 100 µl of samples were injected to a Superdex 200 10/300GL column attached to a Shimadzu chromatography system with inline Wyatt DAWN 8+ and T-rEX detectors. ASTRA software was used for data analysis.

### CD spectroscopy

Protein samples were diluted into 20 mM, KH_2_PO_4_ at pH 6.5 before readings were taken. Protein concentrations were between 0.17 and 0.27 mg/ml. CD spectra were acquired on a JASCO J-815 spectrometer using 1 mm path length quartz cuvettes (Starna Scientific). Readings were taken at intervals of 0.5 nm. Thermal melts were acquired at 207 nm and fit to a variable slope agonist-response curve in Prism 10.

### Intrinsic tryptophan fluorescence measurements

Intrinsic protein fluorescence was measured using a Fluoromax-4 (Horiba) and a High Precision cuvette with 2 × 10 mm path length (Hellma Analytics). An excitation wavelength of 280 nm was used for the total fluorescence measurements. The emission spectra for all samples were recorded from 300 to 400 nm. Intrinsic tryptophan fluorescence measurements used a 295 nm wavelength for excitation and emission was recorded in the range 300–400 nm. All protein samples used for fluorescence experiments were diluted into 20 mM KH_2_PO_4_ at pH 6.5 before taking the measurements, and the final protein concentration in all samples was ∼ 0.2 mg/ml.

### Blue Native PAGE

Yop1 and variant protein samples were run on a NativePAGE 4-16% Bis-Tris Gel (Thermo Fisher Scientific), using 1x NativePAGE buffer as anode buffer and the same buffer with 1x Cathode Buffer Additive as the cathode buffer. Protein samples were ∼0.8 mg/ml and mixed with 1x sample loading buffer before loading into wells. The gel was run at a constant voltage of 150 V at 4°C.

### MD simulations

Starting structure for the Yop1 dimer was generated using AlphaFold-multimer [20]. This structure was embedded in a DOPC bilayer using CHARMM-GUI [29,30]. The system was solvated with TIP3P water and ionized with 0.15 M of NaCl. The standard CHARMM-GUI protocol for minimization and equilibration was run [31]. Next, restraints from NMR experiments in the φ and ψ dihedral angles of the amino acids were applied, with a force constant of 1000 kJ/mol, during a 50 ns simulation. Then, the restraints were removed, and a short simulation of 20 ns was run. This was taken as the starting point for the final 400 ns simulations. Mutations were introduced in this equilibrated wildtype structure using PyMOL (The PyMOL Molecular Graphics System, Version 2.0 Schrödinger, LLC). For the A72E variant, the glutamic acid was assumed to be protonated.

GROMACS 2021.5 [32] was used in all simulations together with the CHARMM36m force field [33]. Production runs were performed using a Nosé-Hoover thermostat [34,35] with a temperature of 303.15 K, with separate temperature coupling for the protein dimers, the membrane, and the solvent and ions, with a coupling time constant of 1.0 ps. The md integrator was used for production runs, with a time step of 2 fs. The pressure was kept at 1 bar using the Parrinello-Rahman barostat [36] with a semi-isotropic pressure coupling scheme, a compressibility of 4.5 × 10^−5^ bar^−1^, and a coupling time constant of 5.0 ps. The Particle Mesh Ewald method was used to compute the electrostatic interactions with a Fourier spacing of 0.16 nm and a cutoff of 1.2 nm. Van der Waals interactions were switched to zero over 1–1.2 nm. The LINCS algorithm was used to constrain bonds involving hydrogen atoms. Periodic boundary conditions were employed in all three directions. Frames were written every 100 ps. Analyses were done using GROMACS tools and considering either the last 100 ns of the trajectories or the entire trajectories. A threshold of 5 Å was taken for all the analysis of contacts. VMD was used for visualization and rendering [37].

## Data Availability

All data supporting the findings of this paper, including computational models, are available upon reasonable request from J.R.S. (jason.schnell@bioch.ox.ac.uk) or S.V. (stefano.vanni@unifr.ch).

## Open Access

Open access for this article was enabled by the participation of University of Oxford in an all-inclusive *Read & Publish* agreement with Portland Press and the Biochemical Society under a transformative agreement with JISC.

## Abbreviations

CD: circular dichroism
dHMN5: distal hereditary motor neuropathy type V
ER: endoplasmic reticulum
LPP: lipoprotein particles
MD: molecular dynamics
SEC: size exclusion chromatography
