# Supplementary material for: Yop1 stability and membrane curvature generation propensity are controlled by its oligomerisation interface
Source: Biochem J. 2024 Oct 11;481(20):1437–48. doi: 10.1042/BCJ20240190 (PMC11555649; doi:10.1042/BCJ20240190)
Supplement: Supplementary Material 1 [file BCJ-481-1437-s1.pdf]

Supplementary Figure 1.

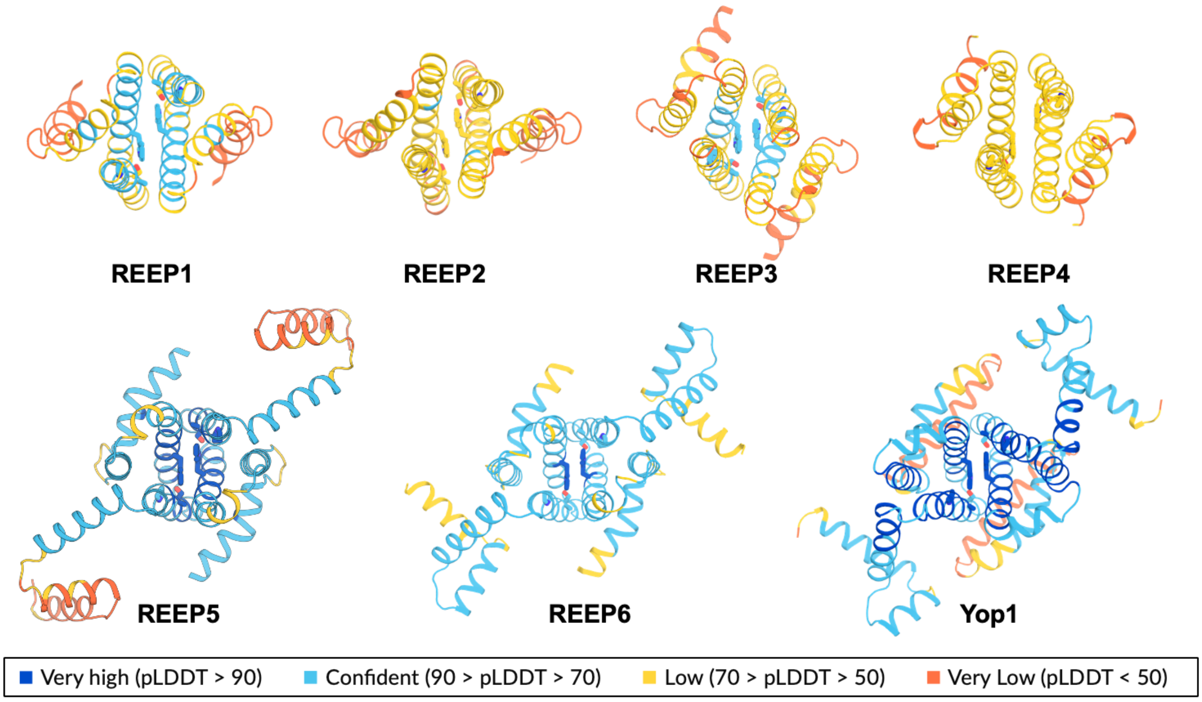

Supplementary Figure 1. AlphaFold-Multimer predictions. AlphaFold-Multimer model predictions for dimeric human REEP1-4, which have three predicted transmembrane domains, and *S. cerevisiae* Yop1 and human REEP5-6, which have four predicted transmembrane domains. For clarity, the unstructured C-terminus after residues 100 were removed for REEP1-4, and the unstructured residues after 148 were removed for REEP5-6.

Supplementary Figure 2.

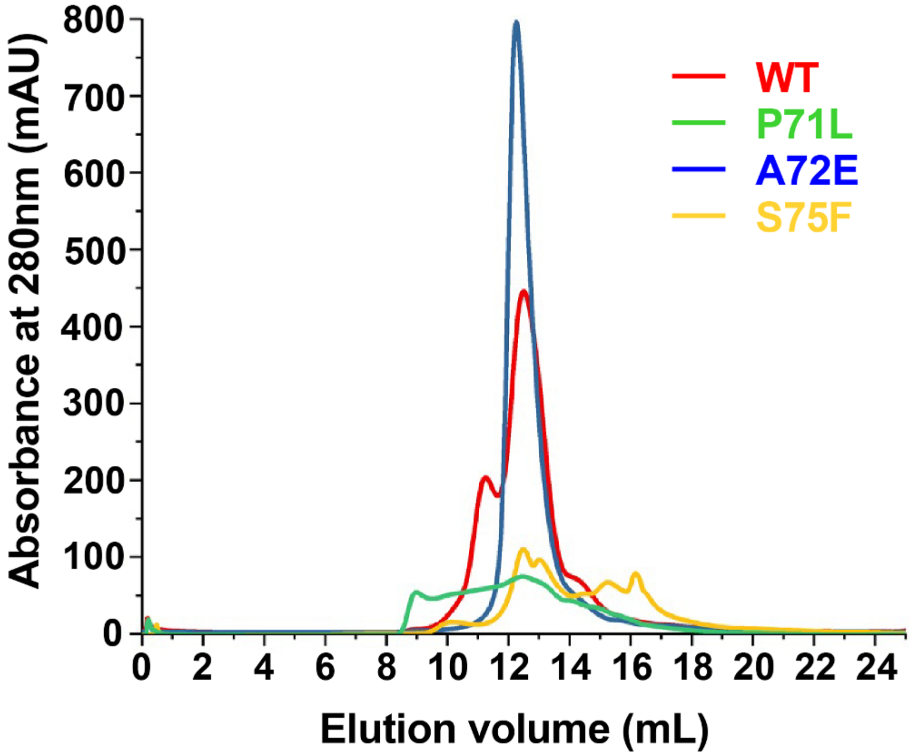

Supplementary Figure 2. Gel filtration purification of full-length Yop1. Example gel filtration chromatograms from the final purification step of Yop1 variants reconstituted into DDM micelles. Samples for experimental studies were obtained from fractions containing the main peaks at ~12-14 ml elution volume.

Supplementary Figure 3.

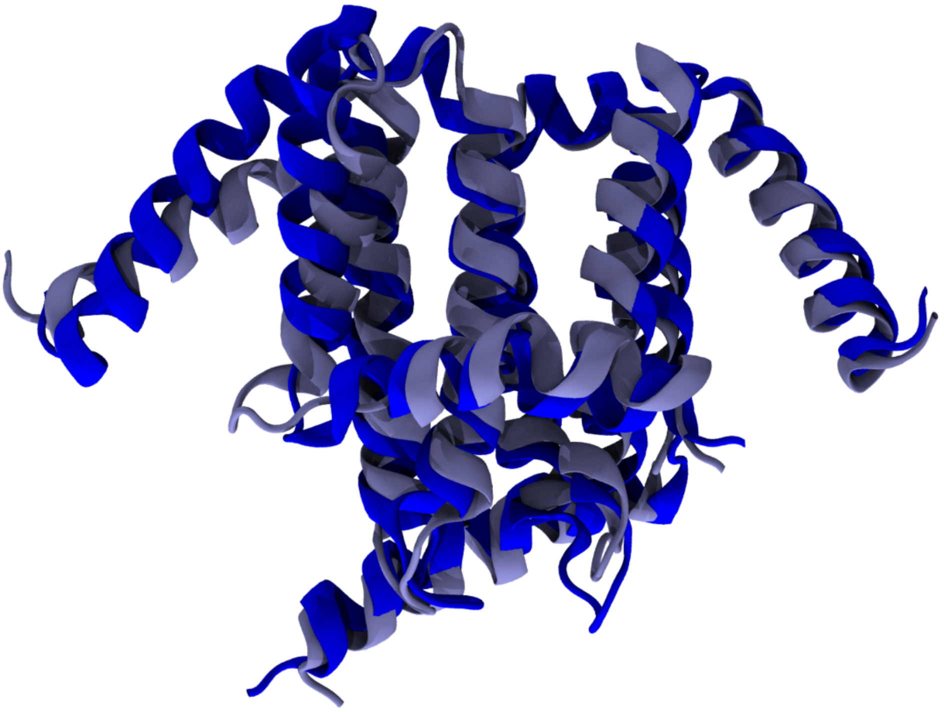

Supplementary Figure 3. Superposition of predicted and refined Yop1 dimer structures. Comparison of the Yop1 dimer model refined against the NMR-derived φ and ψ restraints (Brady et al., 2015) (in blue) with the unrefined model (gray). The RMSD (2.68 Å) was calculated on the backbone of residues from TM1 to the end of the APH.

Supplementary Figure 4.

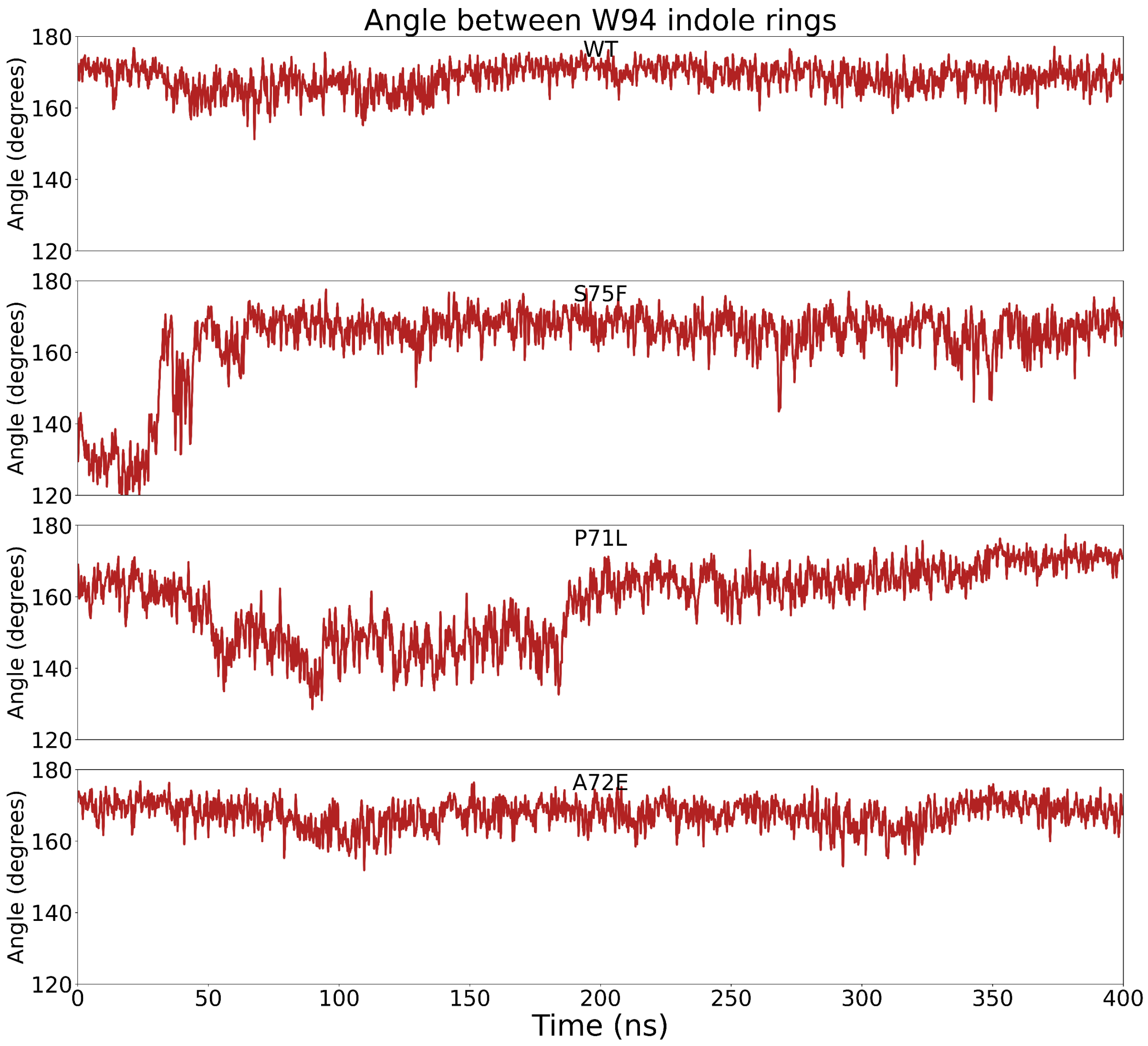

Supplementary Figure 4. Time traces of W94-W94 angles between indole rings for the four Yop1 variants. Values of the dihedral angles between the planes formed by the NE1, CD2, CH2 atoms of each W94 indole ring over time. All variants converge to an almost antiparallel orientation, with values close to 171º.

Supplementary Figure 5.

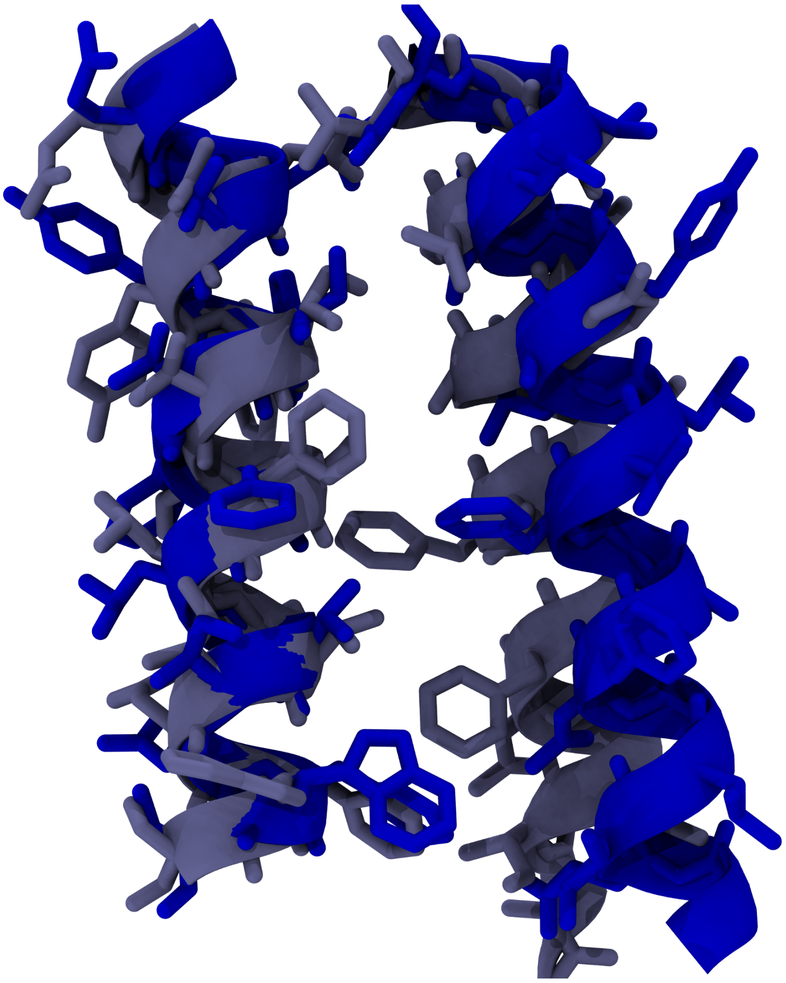

Supplementary Figure 5. Overlap of the two P71L TM2-TM3 interfaces. The blue interface is highly distorted in comparison to the grey one, with larger distances between the sidechains.
